# Short-Chain Fructo-Oligosaccharides Modulate Intestinal Microbiota and Metabolic Parameters of Humanized Gnotobiotic Diet Induced Obesity Mice

**DOI:** 10.1371/journal.pone.0071026

**Published:** 2013-08-12

**Authors:** Frederique Respondek, Philippe Gerard, Mathilde Bossis, Laura Boschat, Aurélia Bruneau, Sylvie Rabot, Anne Wagner, Jean-Charles Martin

**Affiliations:** 1 Innovation, Tereos-Syral, Marckolsheim, France; 2 Aix-Marseille University, Faculté de Médecine, Marseille, France; 3 INSERM, UMR1062 “Nutrition, Obésité et Risque Trombotique”, Marseille, France; 4 INRA, UMR1319 Micalis, Jouy en Josas, France; 5 AgroParisTech, UMR Micalis, Jouy-en-Josas, France; INRA Clermont-Ferrand Research Center, France

## Abstract

Prebiotic fibres like short-chain fructo-oligosaccharides (scFOS) are known to selectively modulate the composition of the intestinal microbiota and especially to stimulate Bifidobacteria. In parallel, the involvement of intestinal microbiota in host metabolic regulation has been recently highlighted. The objective of the study was to evaluate the effect of scFOS on the composition of the faecal microbiota and on metabolic parameters in an animal model of diet-induced obesity harbouring a human-type microbiota. Forty eight axenic C57BL/6J mice were inoculated with a sample of faecal human microbiota and randomly assigned to one of 3 diets for 7 weeks: a control diet, a high fat diet (HF, 60% of energy derived from fat)) or an isocaloric HF diet containing 10% of scFOS (HF-scFOS). Mice fed with the two HF gained at least 21% more weight than mice from the control group. Addition of scFOS partially abolished the deposition of fat mass but significantly increased the weight of the caecum. The analysis of the taxonomic composition of the faecal microbiota by FISH technique revealed that the addition of scFOS induced a significant increase of faecal Bifidobacteria and the *Clostridium coccoides* group whereas it decreased the *Clostridium leptum* group. In addition to modifying the composition of the faecal microbiota, scFOS most prominently affected the faecal metabolome (e.g. bile acids derivatives, hydroxyl monoenoic fatty acids) as well as urine, plasma hydrophilic and plasma lipid metabolomes. The increase in *C. coccoides* and the decrease in *C. leptum,* were highly correlated to these metabolic changes, including insulinaemia, as well as to the weight of the caecum (empty and full) but not the increase in Bifidobacteria. In conclusion scFOS induce profound metabolic changes by modulating the composition and the activity of the intestinal microbiota, that may partly explain their effect on the reduction of insulinaemia.

## Introduction

Due to modern lifestyles that favour the development of excess weight and obesity, type 2 diabetes, which represents around 90% of diabetes cases, is expected to see its prevalence increase by 54% between 2010 and 2030 [1]. Epidemiological studies generally correlate the consumption of high fibre diet with a reduction in the risk of developing type 2 diabetes [2]. A recent meta-analysis has shown that intervention studies with fibres revealed lower fasting blood glucose and glycosylated haemoglobin levels, two markers of glucose intolerance [3]. However it is still not clearly known whether this beneficial effect on the reduction of glycaemic index or energy content, is due to micronutrients associated to fibres or to their fermentation in the colon. It is somehow difficult to evaluate one of these parameters independently from the others in intervention studies, however some recent studies have clearly pointed out a link between the gut microbiota and diet-induced obesity and its associated insulin resistance [4]–[6]. This particularly underlines the possible impact of dietary fibre fermentation in the colon on the regulation of blood glucose and insulin sensitivity of peripheral organs.

Prebiotic soluble fibres like short-chain fructo-oligosaccharides (scFOS) synthesized from beet sugar are known to selectively modulate the composition and the activity of the intestinal microbiota [7]. They will specifically stimulate the development of Bifidobacteria [8], [9] and *Eubacterium rectale* – *Clostridium coccoides* [10], the latter group encompasses most of the butyrate-producing bacteria in the human microbiota [11]. Following an initial increase in lactate production, they induce a higher long-term butyrate production [12], [13] that can be explained by the change in microbial composition. Some studies with inulin-derived ingredients have confirmed that β-fructans also have an effect on other bacterial groups including *Roseburia*, *Faecalibacterium, Eubacterium or Anaerostipes* populations [14], [15].

While the effects of the β-fructans family, to which scFOS belong, on reducing fasting blood glucose in humans are still a matter of debate [16], we have demonstrated that scFOS could improve insulin sensitivity in two animal models of diet-induced obesity [17], [18]. Given that the relationship between the fermentation of food in the colon and insulin sensitivity is generally accepted [5], we can hypothesize that the colonic fermentation of scFOS might be responsible for this effect, highlighting the importance to take the gut microbiota composition into account. While scFOS are well known to stimulate Bifidobacteria in Humans, these bacteria are not always present in animal species where scFOS have shown an improvement of insulin sensitivity. Moreover many other bacterial groups or species have been linked with health effects or patho-physiological states. Therefore we aimed at investigating whether other bacterial groups modulated by the ingredient are correlated with host metabolism by using a humanized mice model. This model provides a reliable microbiota stability over time and can be used to test the effects of different diets, including the addition of fermentable oligosaccharides, on the composition of the microbiota [19], [20]. The effect of scFOS will be particularly studied in a high fat diet known to induce obesity and impair glucose metabolism [21].

In this study, we combined gnotobiology, microbial molecular analysis and metabolomics to decipher the effect of scFOS on the composition of the intestinal microbiota as well as on the metabolic response of the host in a diet-induced obesity (DIO) context.

## Materials & Methods

### Animals and Experimental Design

A fresh stool sample was provided in an anaerobic box (Anaerocult; Merck, Darmstadt, Germany) by a healthy lean adult (40 years, 19< BMI <25) on an unrestricted Western diet, without laxative or antibiotic use for the previous 3 months, who was involved in this study and who gave his/her verbal consent. A 10^−2^ dilution of this stool was administered to 48 germ-free (GF) male C57BL/6J 4-week old mice (initial body weight 20.6±2.9 g) as previously described [19]. As the major components of the human microbiota can be transferred into the ex-germfree animals, these animals are considered the better model to investigate interactions between human intestinal microbiota, host factors and dietary manipulations [19], [20]. Mice were kept in sterile Plexiglas isolators, 3 per cage, at ANAXEM, the GF animal facilities of Micalis Institute (INRA, Jouy-en-Josas, France) for the 10-week duration of the study and had ad libitum access to irradiated feeds and sterile water. Temperature and moisture were carefully controlled. Mice were observed once a day to ensure their welfare. All mice were anaesthetized with isoflurane and then sacrificed by exsanguination. According to the legislation applicable in France, where the whole study was performed, it will only be mandatory as from January 1st 2013 to obtain approval from ethical committee to conduct animal testing, thus it was no applicable when the study was performed. However all researchers were dully allowed to conduct animal testing on laboratory animals and followed the European guidelines on that matter. Furthermore the lab is dully registered under the permission 78–60 at the French Veterinary Services. The adult who provided a faecal sample is one of the author and he gave his verbal consent to do so.

### Diet

After 3 weeks of adaptation, the mice were randomly assigned to one of 3 diets for 7 weeks in a parallel design: a control diet, a high fat diet (HF = 60% of energy from fat) or an isocaloric high-fat diet containing 10% of scFOS (Actilight® 950P, Beghin-Meiji, Marckolsheim, France) (HF-scFOS) (Table 1).

**Table 1:** Composition and nutritional values of the three tested diets.

Ingredient, g/kg (as-fed basis)	Control	HF^1^	HF-scFOS
Casein	195.1	258.4	258.4
L-Cystine	2.9	3.9	3.9
Maltodextrin	312.2	161.5	110.6
Sucrose	341.4	88.9	88.9
scFOS	0.0	0.0	100.0
Cellulose	48.8	64.6	15.5
Soybean oil	24.4	32.3	32.3
Lard	19.5	316.6	316.6
Mineral mix^2^	9.8	12.9	12.9
**Analyzed composition (as fed basis)**			
Crude Protein, g/100 g	19.2	26.2	26.2
Carbohydrates, g/100 g	67.3	26.3	26.3
Fat, g/100 g	4.3	34.9	34.9
Energy, kcal/kg	3.84	5.24	5.24

1 HF = high fat diet, HF-scFOS = isocaloric high fat diet +10% scFOS.

2 Di-calcium phosphate, calcium carbonate, potassium citrate, vitamin mix, choline bitartrate, FD&C.

### Measurements

#### Oral glucose tolerance test

Two days before slaughter, an oral glucose tolerance test (OGTT) was conducted. Blood was collected in EDTA-coated tubes by tail incision after 6 hours of fasting for the initial evaluation of glucose concentration. The animals were then given 2 g of glucose/kg of body weight by oral gavage. Blood glucose was measured again after 20, 40, 60, 90 and 120 min by tail incision on vigilant animals. Insulinaemia was evaluated at 20 and 60 min.

#### Morphological parameters

Body weight and feed consumption were followed weekly throughout the study. Faeces and urine were collected at the beginning and at the end of the study by installing the animals in a metabolic cage for 24 hours. Freshly collected faecal samples were fixed in paraformaldehyde 4% and kept at −80°C until analysis. After 7 weeks of test diet feeding, animals were fasted for 12 hours, re-fed with 1 g of their usual diet and sacrificed 5 hours after this meal in order to collect biological parameters resulting from an adaptation to a high-fat challenge rather than from the resting food-deprived metabolism. A blood sample was taken from the tail vein, body and organs weights were recorded.

#### Glucose, triglycerides and hormone assays

Blood insulin, leptin and glucagon were analysed using the mouse Milliplex kit# MMHMAG-44 K (Millipore, Molsheim, France). Adiponectin was assayed using a mouse ELISA kit# E2MADP-60 K after a 1/100 plasma dilution with saline (Millipore, Molsheim, France). Blood insulin during OGTT was also determined by a mouse ELISA kit# EZRMI-13 K (Millipore, Molsheim, France). Triglycerides were measured with an enzymatic kit#10010303 (Cayman chemical company). Glucose was determined using a glucometer (AccuCheck, Roche diagnostics, Switzerland). All the assays were performed according to the manufacturer’s instructions.

#### Temporal Temperature Gradient gel Electrophoresis analyses

Faecal samples (0.1 g) were used to extract total DNA as previously described [18]. DNA concentration and purity were determined using the Nanodrop ND-1000 spectrophotometer (Thermo Fisher scientific) at wavelength 260/280 nm. The V4/V5 regions of the *Clostridium coccoides* cluster 16S rRNA gene were selectively amplified using primers 5′ AAATGACGGTACCTGACTAA 3′ (forward) and 5′ CTTTGAGTTTCATTCTTGCGAA 3′ (reverse) using the HotStar Master Mix Kit (Qiagen, Courtaboeuf, France). For TTGE analysis of the amplicons, a 40 bp GC-clamp (5′ CGCCCGGGGCGCGCCCCGGGCGGGGCGGGGGCACGGGGGG 3′) was attached to the 5′ end of the forward primer. TTGE was performed as previously described [17] with minor modifications. Briefly, electrophoresis was performed, using the DCode Universal Mutation Detection System (Bio-Rad, Paris, France), through a 1 mm-thick, 16 × 16 cm 10% polyacrylamide gel and run at a fixed voltage of 63 V for 16 h at an initial temperature of 64°C and a ramp rate of 0.3°C/h. The gel was stained in the dark by immersion for 30 min in a solution of SYBR® Gold Nucleic Acid Gel Stain (Invitrogen, Eugene, Oregon) and read on a Storm system (Molecular Dynamics, Bondoufle, France). TTGE band extraction and sequencing were performed as previously described [19]. The sequences obtained were compared to publically available 16S rDNA sequences by nucleotide BLAST (GenBank, NCBI).

#### Fluorescent in situ hybridization analyses

Fluorescent *in situ* hybridization (FISH) combined with flow cytometry was carried out as described previously [22] using a probe panel targeting 9 major bacterial groups of the human gut microbiota: *Clostridium coccoides* group, *Clostridium leptum* subgroup, *Atopobium* cluster, *Bacteroides-Prevotella* group, *Bifidobacterium* genus, Enterobacteria, Erysipelotrichi, *Akkermansia muciniphila*, *Lactobacillus-Enterococcus* group (Table S1). The results were expressed as cells hybridizing with group Cy5 probe as a proportion of the total bacteria hybridizing with the general Eub338 FITC probe. The relationship between these bacterial groups was visualized by hierarchical clustering analysis and mean group values by a heatmap using Permutmatrix software (http://www.lirmm.fr/~caraux/PermutMatrix/) [23] with Pearson correlation square as distance and the Ward method as clustering condition.

#### Metabolomics

All samples of blood, urine and faeces dedicated to metabolomic analysis were frozen immediately and kept at −80°C until further processing.

Urine samples were centrifuged at 17500 g for 10 min at 4°C and then diluted with ultra pure water (1∶4 v/v) and centrifuged again at 800 g for 5 min. The supernatant was collected and kept at −80°C until analysis.

Plasma was thawed before phase partition extraction. Both non-polar compounds and polar compounds were extracted using Bligh and Dyer’s method: 100 µL of plasma was added to 80 µL of saline and 200 µL of methanol. The mixture was vigorously shaken for 30s; 200 µL of chloroform was then added and mixed. After centrifugation (2000 g for 10 min at 4°C), the lower phase was taken with a Pasteur pipette and filtered over Na2SO4. The supernatant was re-extracted with 400 µL of chloroform, and the lower lipid phases were assembled and dried under a nitrogen stream. The methanol-aqueous upper phase was dried by centrifugation under vacuum. Both extracts were stored under a nitrogen atmosphere at –80°C until analysis, and reconstituted with either 500 µL of H2O containing 0.1% formic acid for the polar compounds, or with 500 µL acetonitrile-isopropanol mixture (5∶2, v/v), 0.1% of formic acid and 1% of ammonium acetate for non-polar compounds. Faecal samples were processed as already described [24]. Samples were mixed with 60 volumes (w/v) of phosphate buffer saline (50 mM, pH 8) and sonicated for 75 min and stirred for 30 min at 70rpm at 4°C before being finally centrifuged at 200 g, 4°C for 10 min. The pH of the supernatant was adjusted to 4.5 with formic acid (1%) to each mL of supernatant. Samples were then purified by solid phase extraction (SPE) with sulphonic acid-bonded silica packing for desalination. The cartridges were preconditioned with methanol and water acidified with formic acid (0.1%). The elution was then performed with methanol before injection in the LC-MS.

The samples were analyzed on an Agilent 1200 RRLC coupled to a Brüker microTOF ESI-hybrid quadrupole-time of flight mass spectrometer (Wissembourg, France). For the non-polar compounds (non-polar plasma), the liquid chromatographic conditions were: autosampler set at 15°C; column EC 100/2 Nucleodur C18 Isis, particle size 1.8 µm (Isis1) (Macherey-Nagel, Les Ulis, France). Solvent A: water +0.1% formic acid +0.1% ammonium acetate 1 M; solvent B: acetonitrile-isopropanol (5∶2, v/v) +0.1% formic acid +1% ammonium acetate 1 Meq with 60% B (40% A), 0–5 min 60–100% B, 5–10 min 100% B, 10–12 min 100–60% B, 12–15 min 60% B; flow 0.4 mL/min at 50°C. For polar compounds (plasma aqueous-methanol extracts urine, and faecal samples), the liquid chromatographic conditions were: autosampler set at 4°C; column, EC 100/2 Nucleodur C18 pyramid, particle size 1.8 µm (Macherey-Nagel, Les Ulis, France); started from solvent A (95% water, 5% acetonitrile, 0.1% formic acid) to solvent B (95% acetonitrile, 5% water, 0.1% formic acid) from 0–10 min at 0.4 mL/min at 40°C column oven; thereafter switched to 95% A to 5% B for 6 min, held 2 min, and then returned to 100% A for 5 min. Prior to analysis, the Time of Flight analyzer was calibrated with a sodium formiate solution (*m/z* range from 91 to 1122, HPC and quadratic algorithm, 32 masses used, over 90% accuracy at 0.001% mass window), and a calibration was automatically performed at the beginning of each analysis. The mass spectrometry (MS) conditions were as follows: full scan mode from m/z 50 to m/z 1500; capillary kV, 4.5; capillary temperature, 250°C; cone voltage at 40 V; drying gas flow set at 9.5 L.min-1 and nebulising gas pressure (nitrogen) at 2.4 bar; positive electrospray ionization mode (ESI+).

Raw data files were converted into netCDF format prior to deconvolution with XCMS ran in the R environment [25], [26]. The centwave algorithm was used for feature detection, and obi-warp for retention time peak alignment. Unreliable peaks were removed using a coefficient of variation cut off ≤20% in quality control samples and applied to all samples. Further peak redundancy removal (de-isotoping and de-adduction) was carried out by auto-correlating intensity feature values across quality control samples into the pc group windows calculated by the CAMERA open source package, identifying adducts and isotopes occurring from the parent molecular ions.

The mass spectrometry features retained after statistical analysis were then converted into elemental chemical formula using Data Analysis (Brüker Daltonics, Bremen, Germany), based on their exact mass at about 10000 of mass resolving power, their isotopic pattern (sigma-fit cut off ≤20 at Δppm ≤15) and the adducts formed. Matching elemental chemical formula to molecular structure was performed through the web interface MZedDB allowing simultaneous databases repository requests [27]. The list obtained allowed curation of non-biological or non-metabolites MS feature. This pipeline corresponds to level 2 of the proposed minimum standards for chemical analysis, corresponding to putatively annotated compounds (e.g. without chemical reference standards, based upon physicochemical properties and/or spectral similarity with public/commercial spectral libraries) [28].

### Statistical Analysis

#### Univariate analyses

Differences between groups were analysed by one-way ANOVA followed by Bonferroni’s post hoc multiple comparison test. Calculations were performed using the Statview® software (version 5.0; SAS Institute, Cary, NC, USA). A value of *P*<0.05 was regarded as statistically significant.

#### Multivariate analyses

All multivariate data analyses and modelling were performed using SIMCAP+12 (Umetrics, Umea, Sweden) and were based mainly on principal component analysis and partial least squares methods. Calculations were performed on log10-transformed and pareto-scaled data. A principal component analysis of the pooled data with the quality control samples revealed that the analytical variance was satisfactory low and much lower than the biological variance. All the models evaluated were tested for over fitting, as described previously, with methods including permutation tests, cross-validation and cross validation-ANOVA [26], [29], [30].

**Figure 1:**
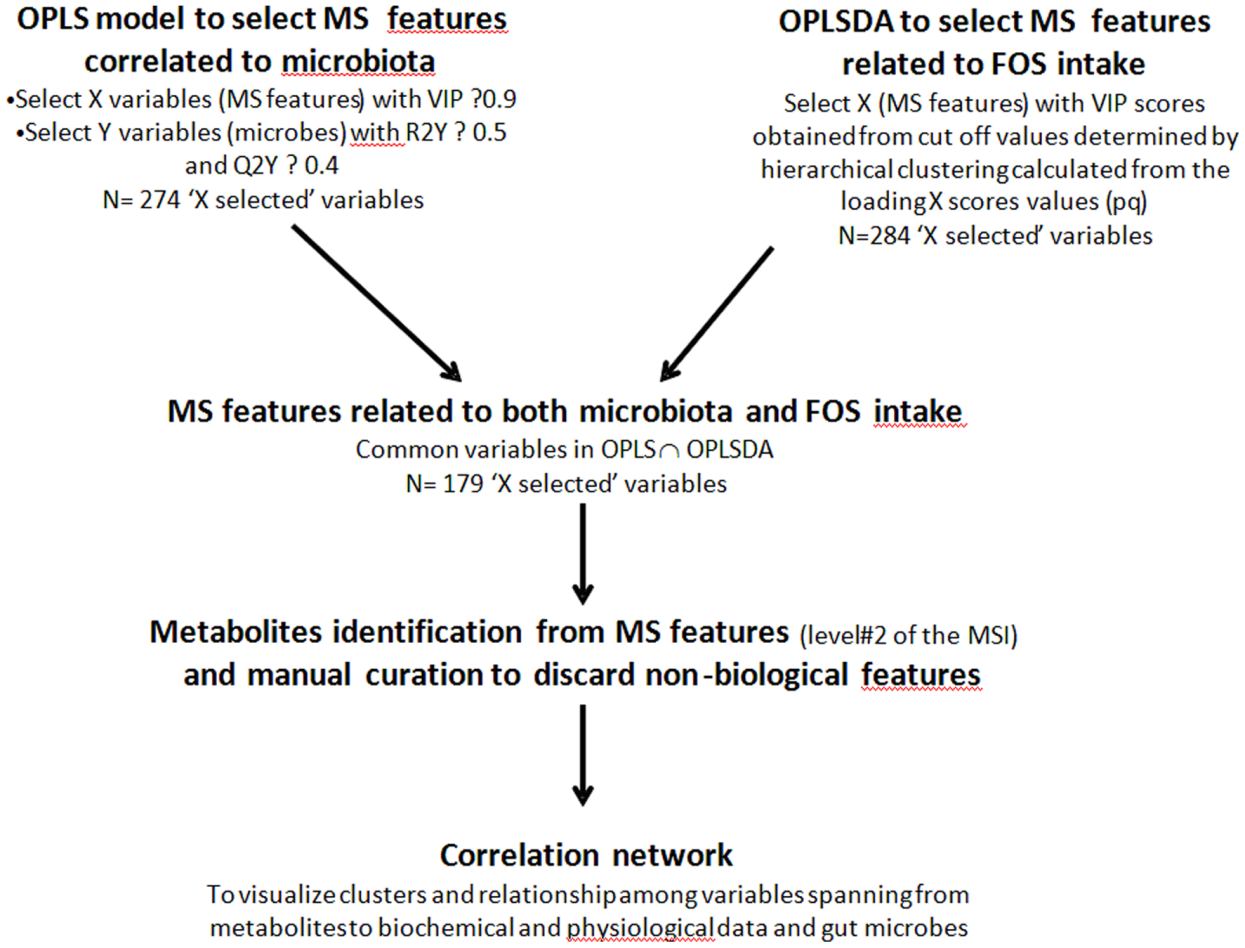
Workflow of the data analysis.

The MS metabolomics data from faeces, urine and plasma were merged into a single dataset along with the corresponding other biological data and microbiota measurements, giving rise to 1804 variables per mouse. One of our primary goals was to find metabolic variables that were associated to the microbiota, affected by scFOS treatment and possibly related to insulin response with an integrated perspective. The flowchart of multivariate statistical analysis to achieve this goal is shown in Figure 1. We first correlated the MS metabolomics features with the microbiota composition in each mouse using an orthogonalized partial least squares analysis. From that analysis, we selected the X variables (metabolomics features) and Y variables (bacterial groups) that were the most associated. The X variables were selected based on a variable importance score cut off ≥0.9 and Y variables using a R2Y value of explained and predicted variance ≥0.5 and ≥0.4, respectively (Figure 1). These thresholds correspond to accepted values for VIP [31] and for the median for R2Y, typically ranging from R2Y = 0 (no fit) to R2Y = 1 (perfect fit). In a second independent step, we determined which metabolomics features were most sensitive to scFOS treatment using an orthogonalized partial least squares discriminant analysis with treatment group used as Y dummy variables (Figure 1). A hierarchical clustering analysis (Ward’s method) was performed on the ‘pq’ score values using the OPLSDA model, to identify which X metabolomics variables (p scores) were most closely associated to the Y group variables (q scores). By crossing the clusters with the variable importance scores, a VIP threshold ≥1.3538 can be defined which selected a subset of X variables very sensitive to dietary treatments. Then, the X variables that were both related to the selected bacterial groups (OPLS model) and to scFOS treatment (OPLSDA model) were retained using a Venn plot (http://bioinfogp.cnb.csic.es/tools/venny/index.html) (Figure 1). The interaction among the various variables spanning from metabolites in the different compartments (faeces, urines and plasma), biochemical and physiological data as well as bacterial strains was visualized using a correlation network (Pearson correlation coefficient threshold at ρ = 0.7) built using Cytoscape open source freeware [32].

## Results

### Analysis of the Microbiota

**Figure 2:**
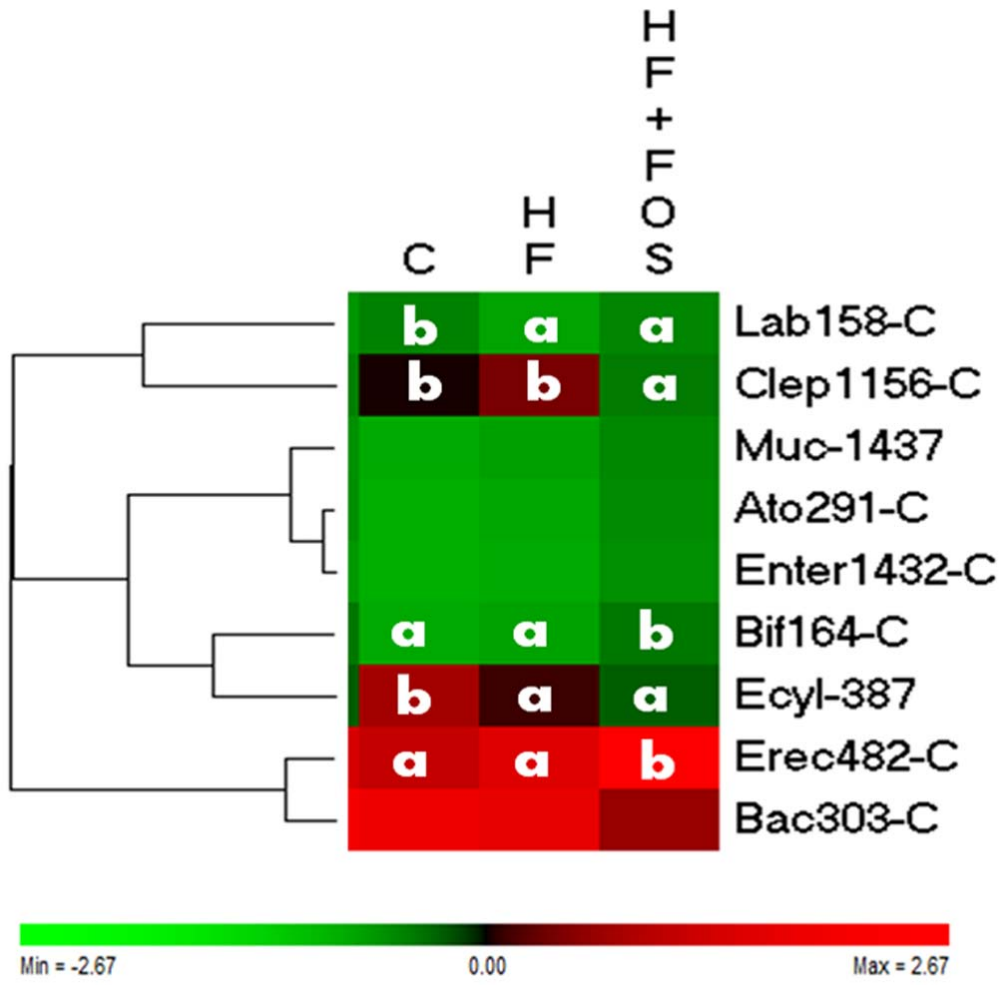
Composition of the faecal microbiota of human microbiota-associated mice after 7 weeks of feeding. The heatmap represents the relative presence of the different bacterial groups when mice received either the control (C) or the high-fat (HF) or the isocaloric high-fat containing 10% of scFOS (HF-scFOS) diets (dark box, increase; light box decrease). A different letter in each row indicates statistically significant difference (Fisher PLSD post-hoc test after ANOVA). Lab 158-C: Lactobacillus - Enterococcus group; Clep 1156-C Clostridium leptum group; Muc 1437 Akkermansia muciniphila species; Ato291-C: Atopobium cluster; Enter 1432-C: Enterobacteria; Bif 164-C: Bifidobacterium genus; Ecyl-387: Erysipelotrichi group; Erec 482-C: Clostridium coccoides group; Bac303-C: Bacteroides – Prevotella group.

The composition of the faecal microbiota was very homogenous among the 3 groups of mice 3 weeks after introduction of human microbiota. *Bacteroides – Prevotella* and *Clostridium coccoides* were the most predominant groups in mice harbouring a human-type microbiota as they accounted for 26±6% and 19±6% of the total bacteria with the control diet, respectively. *Erysipelotrichi* represented 15±10% of the microbiota followed by *Clostridium leptum* at 8±3%. Bifidobacteria accounted for less than 1% of the total bacteria. After 7 weeks of feeding a control, high fat or high fat with scFOS diet the taxonomic composition of the faecal microbiota significantly differed among the 3 groups of mice (Figure 2). The *Lactobacillus-Enteroccocus* (P<0.001) as well as the *Erysipelotrichi* (P<0.01) groups were less represented in the 2 high fat diets than in the control one. The faecal microbiota of mice that received scFOS in their diet presented more Bifidobacteria (P<0.05) and *C. coccoides* (P<0.001) group but less *C. leptum* group (P<0.001) than the one of mice receiving the 2 diets without scFOS (control or high fat). The ratio of *Bacteroides – Prevotella* group: *C. coccoides* group was significantly lower when mice received scFOS (data not shown; P<0.05).

*C. coccoides*-specific TTGE analysis revealed that 2 bands were only present in the faecal microbiota of mice receiving scFOS (Figure S1). These bands were isolated from 3 different lanes; the DNA was extracted and amplified. Sequence analysis indicated that they correspond to *Ruminococcus torques* and *Dorea longicatena*.

### Morphological & Classical Biological Measurements

Mice fed with the 2 high-fat diets displayed higher body weight than mice from the control group even if mice that received the HF-scFOS weighted less than the ones receiving the HF diet (Table 2). Feed intake expressed in g/day and calculated by cage, was greater with the 2 high fat diets than with the control one. However mice fed with the HF-scFOS diet ate less than mice receiving the HF diet. High-fat feeding induced a greater fat mass that was partially abolished by scFOS. Muscle mass was heavier with the HF diet than with the 2 others, whereas mice receiving scFOS showed heavier empty and full caecum than the others (Table 2).

**Table 2:** Body weight and body composition of mice after 7 weeks of feeding a control or high fat diet with or without scFOS.

	Control	HF^1^	HF-scFOS
Body Weight (g)	22.3±1.4 ^a2^	27.1±3.7^ c^	25.0±2.3^ b^
Average feed intake (g/d) 3	2.84±0.4^ a^	3.83±0.8^ c^	3.16±0.5^ b^
Liver (g)	0.96±0.17	1.01±0.18	0.94±0.09
Epididymal fat mass (g)	0.26±0.08^ a^	0.66±0.55^ b^	0.43±0.21^ ab^
Inguinal fat mass (g)	0.11±0.05	0.25±0.24	0.17±0.13
% fat/body weight	1.66±0.42^ a^	3.13±1.9^ b^	2.30±1.07^ ab^
Muscles (g)	0.13±0.02^ a^	0.15±0.01^ b^	0.14±0.02^ a^
Full caecum (g)	0.26±0.06^ a^	0.31±0.10^ a^	0.54±0.11^ b^
Empty caecum (g)	0.10±0.01^ a^	0.11±0.03^ a^	0.17±0.02^ b^

1 HF = high fat diet, HF-scFOS = isocaloric high fat diet +10% scFOS; Data are means ± SD (n = 12/treatment).

2 Within a row, values not sharing the same letter are different (p<0.05).

3 Average feed intake over the whole 56 days of the study.

Among parameters analysed in blood at sacrifice that occurs 5 hours after the last meal, insulin and glucagon did not differ among groups whereas triglycerides were greater with high fat than control feeding. Adiponectin was significantly lower in the HF-scFOS group versus the control one and leptin was also lower in the HF-scFOS group than in the 2 others (Table 3).

**Table 3:** Postprandial blood parameters measured at slaughter of mice fed a control or high fat diet with or without scFOS.

	Control	HF^1^	HF-scFOS
Insulin (ng/mL)	0.52±0.35	0.39±0.21	0.50±0.24
Adiponectin (µg/mL)	17.28±8.70^a2^	11.75±6.66^ab^	9.46±2.54^b^
Leptin (pM)	98.40±73.78^a^	102.78±78.32^a^	27.18±20.29^b^
Glucagon (pM)	7.27±2.63	9.62±4.9	9.10±3.22
Triglycerides (mg/L)	296.6±58.1^a^	417.0±54.4^b^	432.2±123.0^b^

1 HF = high fat diet, HF-scFOS = isocaloric high fat diet +10% scFOS, Data are means ± SD (n = 12/treatment).

2 Within a row, values not sharing the same letter are different (p<0.05).

### OGTT

**Figure 3:**
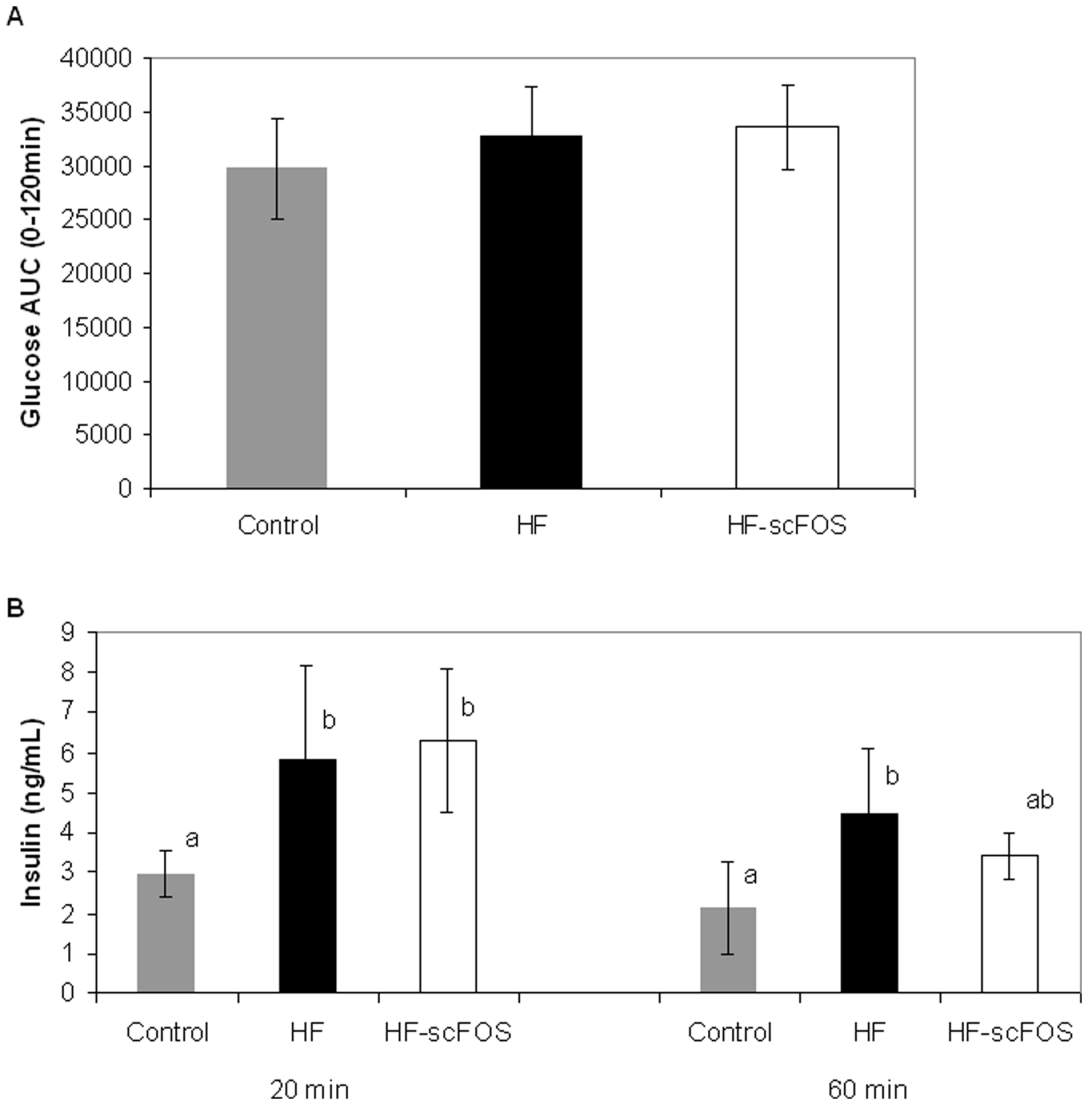
OGTT performed in mice after feeding a control or high fat diet with or without scFOS. A: blood glucose AUC; B: blood insulin sampled at 20 and 60 min after oral load of glucose. ^a,b^ values with different letters are significantly different (p<0.05). HF = high fat diet, HF-scFOS = isocaloric high fat diet +10% scFOS.

The glucose response during the OGTT evaluated as the area under the curve did not differ among the groups but the concentration of blood insulin measured 20 minutes after the glucose load was significantly different between the control and the 2 high fat diets (P<0.05 and P<0.01 respectively for HF and HF-scFOS diets versus the control diet). After 60 minutes, insulin was still higher (P<0.01) but only for the HF diet without scFOS (Figure 3).

### Analysis of the Metabolome

**Figure 4:**
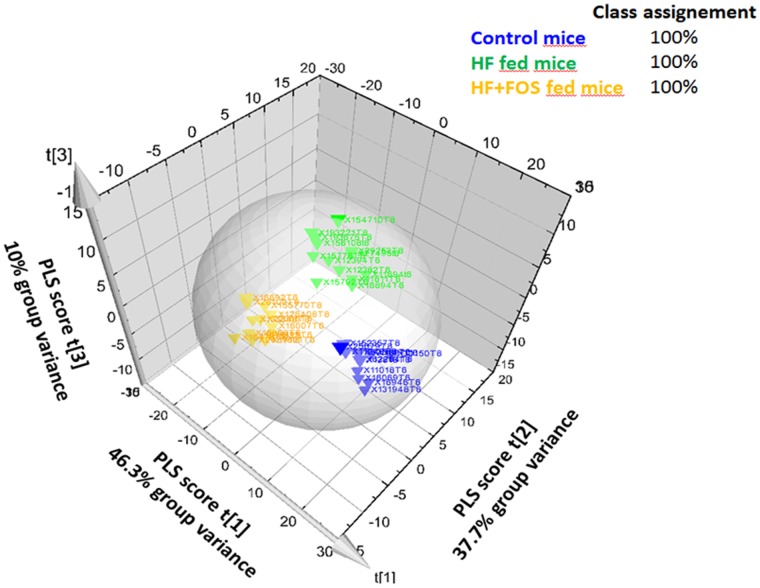
Partial least square discriminant analysis of metabolome of human microbiota-associated mice fed a control or high fat diet with or without scFOS. Class assignment was 100% using the classification list algorithm of SIMCA. Group variance explained R2Y = 94% of observed variance, Q2Y = 78% of predicted variance; the parameters of model validation were: Mean root squares of standard error estimation (RMSEE) = 9.55%; *P* value after CV-ANOVA = 3.24664e-008 (correlation on 65% of total variance). Mean R2Y and Q2Y shifted after 20 permutations at intercept from 0.962 to 0.68 and from 0.79 to −0.32, respectively; all indicators showed therefore good model validation. HF = high fat diet, HF-scFOS = isocaloric high fat diet +10% scFOS.

The time effect on the metabolome can be visualized on the control group mice as a moderate shift on the PCA plot between t0 and t7weeks (Figure S2). Conversely, this time effect was overtaken by feeding the HF diet and most noticeably the HF-scFOS (Figure S2). As a result, the discriminated analysis performed at 7 weeks (OPLSDA) clearly separated the metabolomes (faeces + urine + plasma) according to the 3 different diets (P = 3.25*10^−8^ after CV-ANOVA, after 20 permutations mean % R2Y decrease = 31.05, and mean Q2Y values at intercept = −0.32), indicating that each diet had a different impact on the metabolic profiles (Figure 4).

**Figure 5:**
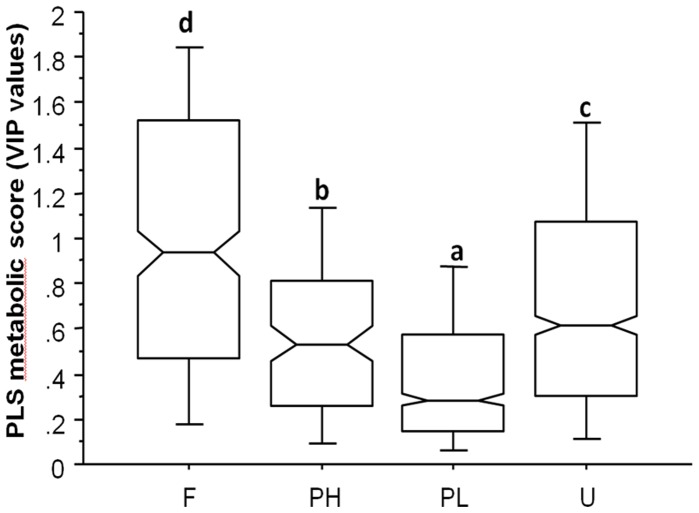
Box and Whiskers plot comparing the composite metabolic scores. VIP values of the n = 1710 metabolic features calculated from the partial least square discrimant analysis of figure 4) for faecal (F), hydrophilic plasma (PH), lipophylic plasma (PL) and urine (U) sample. Each box is delimited by the first quartile Q1 (lower limit of the box), the mediane (second quartile Q2) and the third quartile Q3 (upper box limit). The whiskers represent the adjacent values to the interquartile difference. **a, b, c**, d indicate a significant difference among the groups (Bonferroni/Dunn post-hoc test after ANOVA).

By examining the addition of scFOS to the HF based-diet using a discriminant analysis between HF and HF-scFOS mice at the metabolomics level, we found 152 metabolic features that were highly significantly influenced by scFOS (given by the VIP significant values ≥1.75 selected after hierarchical clustering of the discriminant analysis loadings). Based on their VIP scores, we found that the faecal metabolome was most prominently affected by scFOS treatment, followed by urine and then plasma hydrophilic and finally plasma lipids metabolomes (All *P* values <0.05 after ANOVA, Figure 5). The top 5 metabolites most affected by scFOS (i.e. with the highest VIP values) were hydroxylated fatty acids and bile acids in the faeces, and methyl-hippurate or phenylacetyl-glycine and vitamin B2 and B5 in urine (Table 4).

**Table 4:** Top 5 metabolic features responsible of difference between HF and HF-scFOS diets (OPLSDA analysis).

Metabolome	Exact mass retention time (s)	Corresponding Ion type	Sigma fit - δppm	Chemical formula	Metabolite identification	Increase with diet
Faeces	298.250 (559)	[M+Na]^+^	5.8–10.4	C18H34O3	Hydroxylated C18 fatty acid	HF-scFOS
Faeces	283.262 (539)	[M+H-H2O]^+^	5.1–3.5	C18H36O3	Hydroxylated C18 fatty acid	HF-scFOS
Faeces	391.234 (311)	[M+H-H2O]^+^	1–0.1	C24H40O5	Biliary acid	HF-scFOS
Faeces	263.236 (491)	[M+H-2H2O]^+^	6.4–7.3	C18H34O3	Hydroxylated C18 fatty acid	HF-scFOS
Faeces	293.209 (483)	[M+H-H2O]^+^	5.2–3.8	C16H30O3	Hydroxylated C16 fatty acid	HF-scFOS
Urines	194.081 (269)	[M+H]^+^	1.6–0.8	C10H11NO3	Methyl-hippurate or phenylacetyl-glycine	HF
Urines	203.110 (338)	[M+H-NH3]^+^	28.4–16.1	undertermined	Undetermined	HF
Urines	377.146 (287)	[M+H]^+^	3–5.5	C17H20N4O6	Riboflavine (vitamin B2)	HF-scFOS
Urines	186.113 (325)	[M+H]^+^	4–4.8	undetermined	Undetermined	HF-scFOS
Urines	220.117 (72)	[M+H]^+^	9.2– 0	C9H17NO5	Pantothenic acid (vitamin B5)	HF-scFOS

### Correlation between Biological Parameters, Metabolome and Microbiota

In order to find which bacterial groups were the most strongly related to biological changes upon HF and scFOS feeding, we performed a sequential statistical filtering (Figure 1) that discards the biological response not associated to both bacterial changes in the gut and the dietary (scFOS) effect.

**Figure 6:**
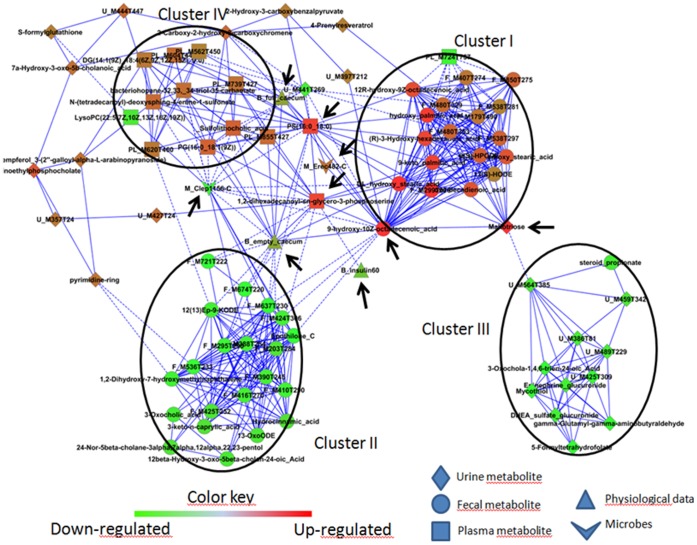
Bacterial group correlation network with metabolites and phenotypic measures (n+1+1) of mice fed high fat diet with or without scFOS. Correlation plots indicating pair-wise correlations among metabolites and bacterial group or phenotypic measures that have a Pearson correlation coefficient value over or equal to 0.7. Node shapes represent the metabolic feature origin as in the legend. Red nodes represent measures that are increased with scFOS and green nodes represent measures that are lowered with scFOS. The edges with solid lines represent positive correlation coefficients and the edges with dashed lines represent negative correlation coefficients. Nodes with an arrow indicates variable of interest (Hub or physiologically relevant variable).

We also performed a pair-wise correlation analysis to show the relationship between the faecal bacterial groups modified by scFOS supplementation and metabolites or phenotypic measures in comparison to the high-fat diet without scFOS (Figure 6). At the cut off level of the Pearson correlation value of 0.7 (giving rise to a minimum determination coefficient ρ^2^ of 0.5 among variables), of the initial 90 variables that were retained after statistical filtering out and data curation, 84 were finally included in the interaction network, among these 51 were fully (biochemical, bacterial and physiological variables) or tentatively annotated (metabolites MS features, level 2 of the minimum standards for chemical analysis) (Table S2). It is of note that the path that linked the insulin response (insulin response at 60 min of the OGTT) to both *C. coccoides* (Erec-482-C; negative link) and *C. leptum* (Clep-156-C; positive link) was very short (through only 1 or 2 plasma lipid metabolites). Four majors clusters can be distinguished in the interaction network, as also calculated by hierarchical clustering analysis (Figure S3 ): one cluster with decreased metabolite content in the faeces upon scFOS feeding (cluster II), containing mainly unknown compounds but some bile acids derivatives and some oxy-fatty acids. This cluster was negatively linked through one C18 hydroxy monoenoic fatty acid to a cluster of up-regulated metabolites comprising many hydroxylated fatty acids (cluster I). These C18 hydroxy-monoenoic fatty acids were directly related to the increase in *C. coccoides* and negatively to *C. leptum* (Figure 6). Apart from these faecal hydroxylated lipids, cluster I also contains an urinary oside molecule which probably corresponds to maltotriose, negatively linking the faecal metabolome to an urinary metabolome cluster (cluster III) (Figure 6). A plasma cluster made up mostly of lipid metabolites was also directly and negatively associated to *C. leptum* (cluster IV). A bacterial sterol (bacteriohopane) was found among the plasma lipids. It is of note that only few variables statistically connected the 4 clusters altogether and thus had hub positions within the network: *C. leptum*, empty and full caecum weight, a urinary oside and a non-identified urinary metabolic feature, a hydroxy-monoenoic faecal fatty acid and 2 plasma phospholipid molecules.

## Discussion

The objective of this study was to correlate some changes induced by scFOS on the composition and activity of the gut microbiota to metabolic parameters at systemic level in an animal model of diet-induced obesity. These humanized gnotobiotic mice harbour a microbiota with the same composition and metabolic activities as a human microbiota that is rather stable over time to allow testing the diet effects, including the addition of fermentable oligosaccharides [20], on the composition of the microbiota.

As expected, the taxonomic composition of the faecal microbiota of gnotobiotic mice evaluated by the FISH technique was similar to that observed in humans with an equivalent technique [33] or with qPCR [34] and was not dramatically altered by the high fat diet except for a slight reduction in the *Lactobacillus-Enterococcus* group and in *Erysipelotrichi*. The reduction of Lactobacilli concentration had already been observed in normal mice receiving a high fat diet and negatively correlated to weight gain and to transepithelial resistance of the proximal colon [35], [36]. Conversely, the introduction of more fat and sugars in the diet had been previously found to increase the proportion of *Erysipelotrichi* in humanized gnotobiotic mice [37]. We can consider whether the difference in sucrose content and/or the quality of fat could explain the difference; but we cannot rule out the possibility that it is due to the different human microbiota used for inoculation.

The composition of the faecal microbiota was strongly modified by the addition of scFOS. As previously observed in humans [8] and in an in-vitro fermentation study with human faecal inoculate [10], scFOS stimulated the growth of Bifidobacteria in humanized gnotobiotic mice. However Bifidobacteria are not predominant in the faecal ecosystem and do not seem to be strongly correlated with metabolite changes in faecal water, urine or blood. More interestingly, scFOS increased the *Clostridium coccoides* group and reduced *Clostridium leptum,* as had been previously demonstrated for another type of β-fructans in human microbiota-associated [14] or obese [38], [39] rats and also in an in-vitro fermentation study using human faecal inoculate [10]. This also confirms a previous study showing that the majority of cultivable inulin-utilizing species, isolated from oligofructose-fed rats, belong to the *C. coccoides* group [40]. The strains particularly stimulated by scFOS within the group of *C. coccoides* were *Ruminococcus torques* and *Dorea longicatena. Ruminococcus torques* is a mucin-degrading bacterium in humans [41]. The presence of this bacterial species is directly and positively correlated with the presence of *Ruminococcus sp. SR1* and *Dorea formicigenerans* [42].

*Dorea longicatena* is a strict anaerobic bacterium with ability poor capacity to use long chain β-fructans, i.e. inulin [43]. It has been identified as a component of the human intestinal microbiota phylogenetic core suggesting that this bacterial species may be essential for a healthy microbiota homeostasis [44]. The lack of effect of scFOS on the *Lactobacillus-Enterococcus* group, *Atopobium* cluster and *Bacteroides-Prevotella* group was previously observed *in-vitro* [10] and *in-vivo* [38]. Globally, these results on gnotobiotic mice are in accordance with what has been observed with a supplementation of fermentable ingredients in conventional mice receiving the same high fat diet [35], [36].

As expected, feeding a high fat diet (60% of energy provided by fat) to mice induced an excessive storage of fat, especially epididymal fat, a higher quantity of consumed feed (g) and a higher body weight in the animals even if the difference with the control remains relatively small in comparison to other studies. This could potentially be explained by the type of microbiota that we used for inoculation as it has been recently demonstrated that despite a common genetic background and nutritional status the metabolic phenotype of mice can be influenced by their gut microbiota profile [45]. In parallel to the greater development of fat mass, the regulation of blood glucose was altered in that more insulin was secreted by the animals during the OGTT than with a control diet. The storage of fat at the epididymal level is generally associated with a reduction of circulating adiponectin and an increase in triglycerides and leptin [46]. Although we observed an increase in triglycerides, we did not observe a higher leptin level. This could be possibly explained by the fact that we measured these parameters in postprandial state and not during fasting. It has been shown that leptin secretion increases after a meal and this could have hidden the effect of obesity in our measurements [47]–[49].

Addition of scFOS to the high-fat diet, while maintaining an equivalent energy density and simple sugars content, decreased final body weight and partially abolished the fat mass increase; this may be explained by a lower feed intake. However the scFOS supplementation does not seem to counteract the reduction in adiponectin and the increase in triglycerides. Similarly to studies in other animal models [17], [18], scFOS did not alter the blood glucose concentration during the OGTT but reduced blood insulin recorded at 60 minutes in comparison to the high fat diet. The fact that the level of insulin is lower at 60 min but not at 20 min with the HF-scFOS *versus* the HF diet is likely to be related to intestinal fermentation and is in accordance with some previous studies in humans showing that consuming high levels of dietary fibres 24 hours before a control meal has no impact on glucose response (AUC, Cmax or Tmax) but significantly decreases insulin response after 100 min [50].

Furthermore, the lower insulin concentration is coherent with the lower concentration of blood leptin observed in the postprandial state, as insulin and leptin resistance are often observed in parallel [51]. This reduction of plasma leptin in the postprandial phase already observed in fasting status with different types of β-fructans [52]–[54], might be explained by a lower leptin release both from mesenteric fat cells and especially those in the small intestine, as it was observed in a study with obese rats fed with 5% of scFOS in replacement of sucrose in their diet [54]. It can also be explained by the fact that propionate induces secretion of leptin from adipose tissue [55] through FFAR3 receptor, whereas scFOS fermentation will induce a higher production of lactate for a transient period followed by the production of butyrate [12]. Unfortunately, our untargeted LC-MS metabolomics analysis is not suitable to detect such SCFA occurring from caecum fermentation but this hypothesis is worth examining in further studies.

Similarly to an antibiotic treatment, scFOS slightly reduced insulinaemia in the late phase of the oral glucose tolerance test that could be related to an improved insulin sensitivity as already shown in two previous studies [17], [18]. ScFOS also led to a significant reduction in feed intake and partially abolished the effect of a high fat diet on fat mass proportion, possibly through the involvement of leptin. The novelty of this study is that a correlation was made between some modifications of the microbiota (i.e. modulation of the *C coccoides* group and *C leptum* subgroup) and some phenotypic and metabolic parameters, and especially the faecal and blood concentrations of bile acids in a gnotobiotic model using a complete human microbiota. Some other related observations between the microbiota composition changes and the host metabolic response was already demonstrated. For instance, a study had already demonstrated that modulating a simplified intestinal microbiota composed of 7 bacterial strains isolated from one healthy infant could alter the host energy and lipid metabolism [56]. Dewulf *et al* had also linked the modifications induced on the microbiota of obese women by inulin-type fructans to bacterial-related metabolites. In this situation *Propionibacterium* was correlated to plasma lactate and phosphatidylcholine while *Collinsella* was rather correlated to urine hippurate [57].

The proposed molecular structures reported were embedded in an interaction network calculated from the correlations among the various variables. The correlation network clearly highlighted the metabolic bacterial/host impact and the continuum of the metabolic regulation that occurred from gut bacteria to the host metabotype previously observed elsewhere [56] and also reported here between the faecal and both urine and plasma metabolomes. Due to its hub position in the interaction network, the decrease in *C. leptum* upon scFOS feeding was accompanied by greater metabolic changes than *C. coccoides*, thereby suggesting that at the microbiota level the scFOS metabolic impact might be more strongly mediated by this bacterial group. The plasma insulin response could be associated to *C. leptum* and *C. coccoides* through a short path involving plasma lipids, possibly identified as phospholipids. As we used a targeted approach for microbiota analysis, it is thus possible that we missed correlations between metabolites and undetected bacterial species. Nevertheless, the correlation network calculated at least highlighted the association between the metabolic response and the bacterial groups we focused. This statistical relationship suggests the involvement of gut bacterial balance in the insulin response, and in the present study it could represent one mechanism of action of scFOS on this biological parameter. High throughput sequencing of the microbiota could confirm our observation obtained with targeted approach and further pinpoint the bacterial species belonging to the *C. leptum* and *C. coccoides* groups.

Conversely, the increase in *C. coccoides* due to scFOS led to a lower *Bacteroides*/*C. coccoides* ratio, which has been shown to be increased in diabetic (Type 2) versus healthy populations [58].

In addition, scFOS feeding induced specific changes of the faecal metabolome certainly related to bacterial metabolism, noticeably the production of hydroxy monoenoic fatty acids and oxygenated fatty acids, and the decrease of some bile acid derivatives. It should be noted that the neutral loss (water) observed in the spectra of these compounds was a good indication of such a hydroxylated structure (not shown). Most faecal metabolites increased by scFOS belong to the category of conjugated fatty acids that are known to be produced by lactic acid bacteria inhabiting the gastrointestinal tract and especially Bifidobacteria and lactobacilli [59]. The alteration of bile acids metabolism seen in this study has already been observed upon probiotic stimulation of the gut microbiome [56] and confirms what was suggested by the modulation of transcription of hepatic Peroxisome Proliferator-Activated Receptor α (PPARα) and Farnesoid X Receptor (FXR) target genes observed in rats supplemented with scFOS [60].

Furthermore it was shown in healthy humans that scFOS lower the conversion rate of primary to secondary bile acids by the intestinal microbiota as illustrated by an increased concentration of faecal primary bile acids (e.g. cholic and chenodeoxycholic acid) and a reduction of secondary bile acids (e.g. lithocholic acid) [13]. This pathway would require further investigation as a recent study has shown that diabetics have a higher production of secondary bile acids from their gut microbiota than healthy subjects [61]. In another study, a significant correlation was found between HOMA-IR and unconjugated bile acids as well as between HbAIc and both secondary and unconjugated bile acids [62]. Furthermore bile acids are already known to regulate lipid and glucose metabolism [63] and especially FXR is known to modulate adiposity and peripheral insulin sensitivity in mice [64].

While the capacity of hydrating octadecaenoic acids seems widely distributed among anaerobic intestinal bacteria, it is not clear which microbes are mostly responsible of the variations of their faecal concentration [65], [66]. Such oxygenated fatty acids are generally thought to be very active compounds [67] but almost no report thus far has been published on how they could influence the host metabolic response when produced in the lower intestine. The action of ricinoleic acid, a 10-hydroxy-octadecenoic acid occurring from castor oil, on the intestinal motility through the action of the intestinal prostanoid EP3 receptor [68] opens the perspective of an interaction between hydroxylated fatty acids of bacterial origin and the host. Also, the identity of the ones reported in the present study needs further confirmation, since isomerisation and structure rearrangement of some may occur during the ionization process. The observation that the weight of caecum (full or empty) is highly related to the metabolomic response, whether in the faeces, plasma or urine in the interaction network, is of note. Already known to be increased by scFOS supplementation [54] and certainly indicating a higher rate of fermentative activities of the microbiota, caecum weight appeared as an easy indicator of the wide biological response to scFOS intake.

The identification of the metabolite features detected in an untargeted metabolomic experiment is not a trivial exercise and is not always feasible, since MS signals might be too low to provide fragmentation spectra, or annotation may not exist in the current databases, or real standards might not be available for comparison. Nevertheless, the information provided from the exact mass and the isotopic pattern coupled with the biological information can provide some clues about the chemical class of the compounds detected, and is now aligned with acceptable practice recognized as level 2 in the proposed minimum metadata relative to metabolite identification [69]. We noticed that when the metabolomic results were oriented towards scFOS feeding only and not scFOS feeding and microbiota relationship, some other metabolic features became important in the statistical model, such as vitamin B2 and B5 in the urine. The technology that was used did not allow determining the SCFA in faecal samples, whereas it is well known that their production is altered by scFOS. Therefore, some other aspects of scFOS biological activities should be investigated.

### Conclusions

Although our results may depend on the human gut microbiota used for inoculation and may not be generalized, they give new insights into the effects of scFOS on gut microbiota composition and metabolic activity in relationship with host metabolism in a context of high fat diet. Especially, our highly integrated approach emphasized two bacterial groups, namely *C. coccoides* and *C. leptum*, that were related to host anatomical (caecum size), physiological (insulin response) and metabolic response (plasma and urine metabolome), possibly through microbiota metabolic changes (faecal metabolome) induced by scFOS feeding. These results provide further hypothesis to be confirmed on how these dietary fibres may contribute to prevent metabolic disorders triggered by environment factors like poor dietary habits, e.g improving insulin sensitivity as shown in previous studies. It would be thus interesting to examine the direct involvement of the hydroxy-fatty acids and bile acids derivatives in the host response we measured. The same kind of approach that we implemented in the present mouse study could be also performed in human and could be helpful to study the scFOS biological response.

## Supporting Information

Figure S1 **PCA of the metabolomic data obtained from mice fed the HF, HF-scFOS and control diets, and showing a time and diet interplay.**
(TIF)

Figure S2 **TTGE profiles obtained from mice fed the HF, HF-scFOS and control diets.**
(TIF)

Figure S3 **Calculation of the cluster limits depicted in** **Figure 6** **of the manuscript by hierarchical clustering analysis (Ward's method, pearson correlation square values as distance).** Annotation and cluster assignments of variables are reported in the Table S2
(TIF)

Table S1 **Designations, sequences and targets of probes used in fluorescent** ***in situ*** **hybridization experiments.**
(DOC)

Table S2 **Annotation of the variables shown in** **Figure 6** **of the manuscript.**
(DOCX)
