# Supplementary figures and images for: Short-Chain Fructo-Oligosaccharides Modulate Intestinal Microbiota and Metabolic Parameters of Humanized Gnotobiotic Diet Induced Obesity Mice
Source: PLoS One. 2013 Aug 12;8(8):e71026. doi: 10.1371/journal.pone.0071026 (PMC3741321; doi:10.1371/journal.pone.0071026)

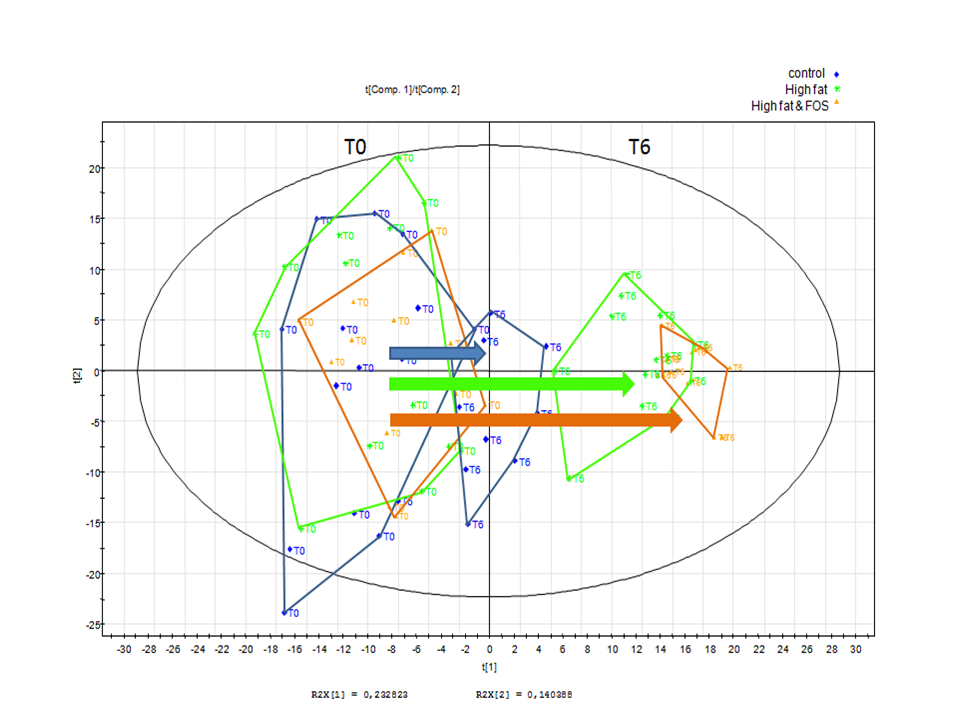

Supplement: Figure S1 — PCA of the metabolomic data obtained from mice fed the HF, HF-scFOS and control diets, and showing a time and diet interplay. (TIF) [file pone.0071026.s001.tif]
