# Supplementary material for: Short-Chain Fructo-Oligosaccharides Modulate Intestinal Microbiota and Metabolic Parameters of Humanized Gnotobiotic Diet Induced Obesity Mice
Source: PLoS One. 2013 Aug 12;8(8):e71026. doi: 10.1371/journal.pone.0071026 (PMC3741321; doi:10.1371/journal.pone.0071026)
Supplement: Table S1 — Designations, sequences and targets of probes used in fluorescent in situ hybridization experiments. (DOC) [file pone.0071026.s004.doc]

Supplemental Table 1: Designations, sequences and targets of probes used in fluorescent *in situ* hybridization experiments

| **Probe** | **Sequence (5’ → 3’)** | **Target** | **Fluorochrome** |
| --- | --- | --- | --- |
| Eub338 | GCTGCCTCCCGTAGGAGT | domain Bacteria | 5’FITC |
| NonEub338 | ACATCCTACGGGAGGC | none | 5’FITC |
| Erec 482 | GCTTCTTAGTCAGGTACCG | *Clostridium coccoides* group | 5’CY5 |
| Clep1156 | GTTTTRTCAACGGCAGTC  Competitor 1 GRTTTRTCAYCGGCAGTC^*^  Competitor 2 GTVTTRTCBACGGCAGTC^*^ | *Clostridium leptum subgroup* | 5’CY5 |
| Ato291 | GGTCGGTCTCTCAACCC | *Atopobium* cluster | 5’CY5 |
| Bac303 | CCAATGTGGGGGACCTT | *Bacteroides-Prevotella* group | 5’CY5 |
| Bif164 | CATCCGGCATTACCACCC | *Bifidobacterium* genus | 5’CY5 |
| Enter1432 | CTTTTGCAACCCACT | Enterobacteria | 5’CY5 |
| Ecyl387 | CGCGGCATTGCTCGTTCA | Erysipelotrichi | 5’CY5 |
| Muc1437 | CCTTGCGGTTGGCTTCAGAT | *Akkermansia muciniphila* | 5’CY5 |
| Lab158 | GGTATTAGCAYCTGTTTCCA | *Lactobacillus-Enterococcus* group | 5’CY5 |
