# Supplementary material for: Short-Chain Fructo-Oligosaccharides Modulate Intestinal Microbiota and Metabolic Parameters of Humanized Gnotobiotic Diet Induced Obesity Mice
Source: PLoS One. 2013 Aug 12;8(8):e71026. doi: 10.1371/journal.pone.0071026 (PMC3741321; doi:10.1371/journal.pone.0071026)
Supplement: Table S2 — Annotation of the variables shown in Figure 6 of the manuscript. (DOCX) [file pone.0071026.s005.docx]

Supplemental Table 2: Annotation of the variables shown in Figure 6 of the manuscript.

| **Origin**^1^ | **Proposed annotation**^2^ | **Molecular formula** | **Adducts** | **Cluster** |
| --- | --- | --- | --- | --- |
| F | [11,12-dihydroxy stearic acid](http://maltese.dbs.aber.ac.uk:8888/hrmet/search/printmet.php?metid=D7533) | C18H36O4 | [M+H-H2O]1+ | I |
| F | [11,12-dihydroxy stearic acid](http://maltese.dbs.aber.ac.uk:8888/hrmet/search/printmet.php?metid=D7533) | C18H36O4 | [M+H-H2O]1+ | I |
| F | [12R-hydroxy-9Z-octadecenoic acid](http://maltese.dbs.aber.ac.uk:8888/hrmet/search/printmet.php?metid=D16105) | C18H35O3 | [M+H-H2O]1+ | I |
| F | [13(S)-HODE](http://maltese.dbs.aber.ac.uk:8888/hrmet/search/printmet.php?metid=D16493) | C18H32O3 | [M+Na]1+ | I |
| F | [13(S)-HODE](http://www.genome.ad.jp/dbget-bin/www_bget?compound+C14762) | C18H33O3 | [M+H]1+ | I |
| F | [9-hydroxy-10Z-octadecenoic acid](http://maltese.dbs.aber.ac.uk:8888/hrmet/search/printmet.php?metid=D15922) | C18H34O3 | [M+Na]1+ | I |
| B | insulin60 | NA | NA | I |
| F | [DL-hydroxy stearic acid](http://maltese.dbs.aber.ac.uk:8888/hrmet/search/printmet.php?metid=D10920) | C18H35O2 | [M+H]1 -H2O | I |
| F | M203T284 | unknown | unknown | I |
| F | M410T290 | unknown | unknown | I |
| F | M480T353 | C27H46NO4S | unknown | I |
| F | M536T233 | unknown | unknown | I |
| F | M538T297 | unknown | unknown | I |
| F | M637T230 | unknown | unknown | I |
| U | [gamma-Glutamyl-gamma-aminobutyraldehyde](http://maltese.dbs.aber.ac.uk:8888/hrmet/search/printmet.php?metid=D26130) | C20H36N5O8 | [[2M+ACN+H]1+](http://maltese.dbs.aber.ac.uk:8888/hrmet/search/isotope.php?molform=C20H36N5O8&charge=1&nelec=-1&rellim=1) | I |
| U | Kaempferol 3-(2''-galloyl-alpha-L-arabinopyranoside) | C_27_H_22_O_14_ | [M+H-H2O]^+^ | I |
| U | [Maltotriose](http://maltese.dbs.aber.ac.uk:8888/hrmet/search/printmet.php?metid=D29327) | C18H32O16 | [M+Na]1+ | I |
| PL | PG(16:0/18:1(9Z)) | [C40H81NO10P](http://www.lipidmaps.org/tools/ms/iso2d_Ag.php?formula=C40H81NO10P) | M+NH4 | I |
| PL | M739T427 | unknown | unknown | I |
| F | [1,2-Dihydroxy-7-hydroxymethylnaphthalene](http://maltese.dbs.aber.ac.uk:8888/hrmet/search/printmet.php?metid=D29505) | C11H10O3 | [M+2ACN+H]1+ | II |
| F | [12beta-Hydroxy-3-oxo-5beta-cholan-24-oic Acid](http://maltese.dbs.aber.ac.uk:8888/hrmet/search/printmet.php?metid=D22545) | C24H37O3 | [M+H-H2O]1+ | II |
| F | 13-OxoODE | C18H30O3 | [M+H-H2O]1+ | II |
| F | [24-Nor-5beta-cholane-3alpha,7alpha,12alpha,22,23-pentol](http://maltese.dbs.aber.ac.uk:8888/hrmet/search/printmet.php?metid=D22328) | C23H40O5 | [2M+Na]1+ | II |
| F | [3-keto-n-caprylic acid](http://maltese.dbs.aber.ac.uk:8888/hrmet/search/printmet.php?metid=D6392) | C8H14O3 | [M+ACN+H]1+ | II |
| U | [3-Oxochola-1,4,6-trien-24-oic Acid](http://maltese.dbs.aber.ac.uk:8888/hrmet/search/printmet.php?metid=D22488) | C24H32O3 | [M+Na-H2O]1+ | II |
| U | [4-Prenylresveratrol](http://maltese.dbs.aber.ac.uk:8888/hrmet/search/printmet.php?metid=D1839) | C19H21O3 | [M+H]1+ | II |
| PL | [bacteriohopane-32,33, 34-triol-35-carbamate](http://maltese.dbs.aber.ac.uk:8888/hrmet/search/printmet.php?metid=D3333) | C36H63NO5 | [M+H]1+ | II |
| U | [DHEA sulfate glucuronide](http://maltese.dbs.aber.ac.uk:8888/hrmet/search/printmet.php?metid=D24030) | C19H28O5S | [M+H]1+ | II |
| F | M179T490 | unknown | unknown | II |
| F | M295T290 | unknown | unknown | II |
| F | M388T254 | unknown | unknown | II |
| F | M390T245 | unknown | unknown | II |
| F | M407T274 | C22H39N4OS | unknown | II |
| F | M416T270 | unknown | unknown | II |
| F | M424T306 | unknown | unknown | II |
| F | M425T252 | unknown | unknown | II |
| F | M480T329 | unknown | unknown | II |
| F | M538T281 | unknown | unknown | II |
| F | M674T220 | unknown | unknown | II |
| F | M721T222 | unknown | unknown | II |
| F | M850T275 | unknown | unknown | II |
| F | [Hydrocinnamic acid](http://maltese.dbs.aber.ac.uk:8888/hrmet/search/printmet.php?metid=D27574) | C9H10O2 | [2M+H]1+ | II |
| F | (R)-3-Hydroxy-hexadecanoic acid | C16H32O3 | [M+H]1+ | III |
| F | [9(S)-HPODE](http://maltese.dbs.aber.ac.uk:8888/hrmet/search/printmet.php?metid=D16609) | C18H32O4 | [M+H-H2O]1+ | III |
| B | full_caecum | NA | NA | III |
| U | Epinephrine glucuronide | C15H21NO9 | [M+K]1+ | III |
| F | [Epothilone C](http://maltese.dbs.aber.ac.uk:8888/hrmet/search/printmet.php?metid=D23483) | C26H39NO5S | [M+2ACN+H]1+ | III |
| F | hydroxy palmitic acid | C16H32O3 | [M+H-H2O]^+^ | III |
| PL | [N-(tetradecanoyl)-deoxysphing-4-enine-1-sulfonate](http://maltese.dbs.aber.ac.uk:8888/hrmet/search/printmet.php?metid=D15040) | [C32H63NO5S](http://maltese.dbs.aber.ac.uk:8888/hrmet/search/isotope.php?molform=C33H68NO6S&charge=1&nelec=-1&rellim=1) | [M+H+CH3OH]1+ | III |
| U | M357T24 | unknown | unknown | III |
| U | M397T212 | unknown | unknown | III |
| U | M427T24 | unknown | unknown | III |
| U | M489T229 | unknown | unknown | III |
| U | M564T385 | unknown | unknown | III |
| PL | [**Sulfolithocholic acid**](http://maltese.dbs.aber.ac.uk:8888/hrmet/search/printmet.php?metid=D22533)^3^ | [**C24H40O6S**](http://maltese.dbs.aber.ac.uk:8888/hrmet/search/isotope.php?molform=C26H44NO6S&charge=1&nelec=-1&rellim=1) | **M+ACN+H]1+** | **IV** |
| PL | 1,2-dihexadecanoyl-sn-glycero-3-phosphoserine | C38H74NO10P | [M+H]1+ | IV |
| F | 12(13)Ep-9-KODE | C18H30O4 | [M+H-2H2O]^+^ | IV |
| U | [2-Aminoethylphosphocholate](http://maltese.dbs.aber.ac.uk:8888/hrmet/search/printmet.php?metid=D21403) | C28H49N2NaO7P | [M+ACN+Na]1+ | IV |
| U | [**2-Carboxy-2-hydroxy-8-carboxychromene**](http://maltese.dbs.aber.ac.uk:8888/hrmet/search/printmet.php?metid=D27803) | **C11H8O6** | [**[M+IsoProp+H]1+**](http://maltese.dbs.aber.ac.uk:8888/hrmet/search/isotope.php?molform=C14H17O7&charge=1&nelec=-1&rellim=1) | **IV** |
| U | [2-Hydroxy-3-carboxybenzalpyruvate](http://maltese.dbs.aber.ac.uk:8888/hrmet/search/printmet.php?metid=D27045) | C11H8O6 | [M+IsoProp+H]1+ | IV |
| F | [3-Oxocholic acid](http://maltese.dbs.aber.ac.uk:8888/hrmet/search/printmet.php?metid=D22429) | C24H38O5 | [M+H]1+ | IV |
| U | 5-Formyltetrahydrofolate | C25H32NO10 | [M+CH3OH+H]^+^ | IV |
| U | [7a-Hydroxy-3-oxo-5b-cholanoic acid](http://maltese.dbs.aber.ac.uk:8888/hrmet/search/printmet.php?metid=D22513) | C24H38O4 | [M+H]1+ | IV |
| F | [9-hydroxy-10Z-octadecenoic acid](http://maltese.dbs.aber.ac.uk:8888/hrmet/search/printmet.php?metid=D15922) | C18H36O4 | [M+H]1+ | IV |
| F | [9-keto palmitic acid](http://maltese.dbs.aber.ac.uk:8888/hrmet/search/printmet.php?metid=D7944) | C16H30O3 | [M+Na]1+ | IV |
| PL | Bacteriohophane-32,33,34-triol-35-carbamate | C36H63NO5 | [M+H]1+ | IV |
| B | empty_caecum | NA | NA | IV |
| B | **Full caecum** | **NA** | **NA** | **IV** |
| PL | [**DG(14:1(9Z)/18:4(6Z,9Z,12Z,15Z)/0:0)**](http://maltese.dbs.aber.ac.uk:8888/hrmet/search/printmet.php?metid=D6292) | [**C35H58O5**](http://maltese.dbs.aber.ac.uk:8888/hrmet/search/isotope.php?molform=C35H62NO5&charge=1&nelec=-1&rellim=1) | **[M+NH4]1+** | **IV** |
| PL | [**LysoPC(22:5(7Z,10Z,13Z,16Z,19Z))**](http://maltese.dbs.aber.ac.uk:8888/hrmet/search/printmet.php?metid=D5792) | **C30H52NO7P** | **[M+H]1+** | **IV** |
| M | Clep1156-C | NA | NA | IV |
| M | Erec482-C | NA | NA | IV |
| U | [Mycothiol](http://maltese.dbs.aber.ac.uk:8888/hrmet/search/printmet.php?metid=D3801) | C17H34N3O12S | [M+NH4]1+ | IV |
| PL | N-(tetradecanoyl)-deoxysphing-4-enine-1-sulfonate | C32H63NO5S | [M+H+CH3OH]1+ | IV |
| F | oxo-decadienoic acid | [M+H]1+ | C18H37O4 | IV |
| PL | **M562T450** | **unknown** | **unknown** | **IV** |
| PL | **PG(16:0/18:1(9Z))** | **C40H81NO10P** | **[M+NH4]1+** | **IV** |
| PL | **M604T449** | **unknown** | **unknown** | **IV** |
| PL | **M620T460** | **unknown** | **unknown** | **IV** |
| PL | M724T757 | unknown | unknown | IV |
| PL | **M739T427** | **unknown** | **unknown** | **IV** |
| PL | **M855T427** | **unknown** | **unknown** | **IV** |
| PL | PS(16:0/18:0) | [C40H79NO10P](http://www.lipidmaps.org/tools/ms/iso2d_Ag.php?formula=C40H79NO10P) | [M+H]1+ | IV |
| U | [pyrimidine-ring](http://maltese.dbs.aber.ac.uk:8888/hrmet/search/printmet.php?metid=D30900) | C4H4N2 | [M+2ACN+H]1+ | IV |
| U | [S-Formylglutathione](http://www.hmdb.ca/metabolites/HMDB01550) | C13H21N4O7S | [M+ACN+H]1+ | IV |
| F | steroid propionate | C24H34O6 | [M+Na]1+ | IV |
| U | M386T81 | unknown | unknown | IV |
| PL | **Sulfolithocholic acid** | **C24H40O6S** | **[M+ACN+H]1+** | **IV** |
| U | M425T309 | unknown | unknown | IV |
| U | M441T269 | unknown | unknown | IV |
| U | M444T447 | unknown | unknown | IV |
| U | M459T342 | unknown | unknown | IV |

^1^B, biological data, F, fecal metabolites, PL, plasma metabolites, U, urine metabolites

^2^ For the non-annotated mass spectrometry features, M indicated the absolute m/z value and T the retention times (s) value of the feature in the XCMS output.

^3^Variables in bold characters represent the core of the figure 6 cluster IV. NA stands for Not Applicable (for molecular formula and adduct forms).
